# Widespread and dynamic expression of granzyme C by skin-resident antiviral T cells

**DOI:** 10.3389/fimmu.2023.1236595

**Published:** 2023-09-21

**Authors:** Ramon A. Lujan, Luxin Pei, John P. Shannon, Nathânia Dábilla, Patrick T. Dolan, Heather D. Hickman

**Affiliations:** ^1^ Viral Immunity and Pathogenesis Unit, Laboratory of Clinical Immunology and Microbiology, National Institute of Allergy and Infectious Diseases (NIAID), National Institutes of Health (NIH), Bethesda, MD, United States; ^2^ School of Nursing, Duke University, Durham, NC, United States; ^3^ Quantitative Virology and Evolution Unit, Laboratory of Viral Diseases, NIAID, NIH, Bethesda, MD, United States

**Keywords:** granzymes, antiviral immunity, unconventional T cells, poxvirus, microscopy

## Abstract

After recognition of cognate antigen (Ag), effector CD8^+^ T cells secrete serine proteases called granzymes in conjunction with perforin, allowing granzymes to enter and kill target cells. While the roles for some granzymes during antiviral immune responses are well characterized, the function of others, such as granzyme C and its human ortholog granzyme H, is still unclear. Granzyme C is constitutively expressed by mature, cytolytic innate lymphoid 1 cells (ILC1s). Whether other antiviral effector cells also produce granzyme C and whether it is continually expressed or responsive to the environment is unknown. To explore this, we analyzed granzyme C expression in different murine skin-resident antiviral lymphocytes. At steady-state, dendritic epidermal T cells (DETCs) expressed granzyme C while dermal γδ T cells did not. CD8^+^ tissue-resident memory T cells (T_RM_) generated in response to cutaneous viral infection with the poxvirus vaccinia virus (VACV) also expressed granzyme C. Both DETCs and virus-specific CD8^+^ T_RM_ upregulated granzyme C upon local VACV infection. Continual Ag exposure was not required for maintained T_RM_ expression of granzyme C, although re-encounter with cognate Ag boosted expression. Additionally, IL-15 treatment increased granzyme C expression in both DETCs and T_RM_. Together, our data demonstrate that granzyme C is widely expressed by antiviral T cells in the skin and that expression is responsive to both environmental stimuli and TCR engagement. These data suggest that granzyme C may have functions other than killing in tissue-resident lymphocytes.

## Introduction

Granzymes are a family of serine proteases that are expressed by both innate and adaptive cytotoxic lymphocytes (1–3). There are 11 granzymes in mice (A-G, K-N) and 5 granzymes in human (A, B, H, K, M). Granzymes A and B have been the most extensively studied of the family, primarily in the context of killing infected or neoplastic cells (4). However, there are several family members, including granzyme C in mice and granzyme H in humans, with currently unknown function(s) (5).

Granzymes are expressed by different innate and adaptive immune cells, with unique cellular populations expressing different granzymes. Granzymes A and B are highly expressed by cytotoxic effector cells, including T cells and natural killer (NK) cells (6). Murine CD8^+^ T cells can also express Granzyme K, which has been associated with inflammatory aging (7). Human CD8^+^ T cells produce granzyme K in inflamed tissues (8). Human NK cells have been demonstrated to translate high levels of granzymes H and M in certain conditions (9, 10). Mouse CD4^+^ and CD8^+^ T cells synthesize granzyme C primarily *in vitro* or in the context of mixed lymphocyte reactions (2, 11, 12). Recently, mouse granzyme C was shown to be constitutively expressed by cytolytic group 1 innate lymphoid cells (ILC1s) in the liver and salivary gland (13). Thus, the unique tissue environments and cellular functions of lymphocytes support the differential expression of granzyme family members.

Mechanistically, granzymes have been shown to function primarily during cytolysis. Cytotoxic lymphocytes recognizing cognate Ag form an immune synapse and directionally secrete granzymes toward target cells along with the pore-forming protein perforin (14). Perforin/granzyme secretion is critical for the control of some viral infections, including ectromelia virus (mousepox) and lymphocytic choriomeningitis virus (LCMV) (15, 16). However, numerous studies have now demonstrated that granzymes possess non-canonical activities as well (6, 17). For example, CD8^+^ T cells and NK cells use granzyme B independently from perforin to extravasate into the tissues and traffic to sites of infection *in vivo* (18). Granzyme B can degrade extracellular matrix proteins and may affect various physiological processes such as basement membrane degradation, collagen disorganization, and wound healing (18–20). Additionally, granzyme A can reach high levels in human serum during infection with human immunodeficiency virus (HIV), Epstein-Barr virus (EBV), and Chikungunya virus (CHIKV) (6). Exogenously produced granzyme K can induce inflammation in non-lymphoid cells such as fibroblasts and activate endothelial cells (7, 21). Thus, granzymes can remodel the tissue environment through their protease activity.

In addition to granzyme C function, the regulation of its expression is also unknown. Some ILC1s constitutively produce granzyme C *in vivo* (13). ILC1s do not express the rearranged Ag receptors of adaptive lymphocytes, suggesting that factors other than Ag recognition might drive granzyme C production. Granzyme C expression may be developmentally hard-wired in some cells, or it may occur in response to changes in the local environment. Here, we sought to understand 1) whether ILC1s are the only tissue-resident lymphocytes that express granzyme C and 2) what factors regulate granzyme C expression. To do this, we analyzed granzyme C expression in different T cell populations present in the mouse skin: activated effector CD8^+^ T cells, tissue-resident memory (T_RM_) CD8^+^ T cells, and γδ T cells. Although the level and frequency of granzyme C expression varied, a percentage of each skin-resident T cell population expressed granzyme C. Interestingly, skin-resident γδ T cells and CD8^+^ αβ T_RM_ expressed granzyme C at steady-state and upregulated granzyme C following primary or secondary infection. Exposure to cognate Ag in the absence of virus-driven inflammation also increased granzyme C expression in CD8^+^ αβ T_RM_. However, a proportion of CD8^+^ αβ T cells maintained granzyme C expression in the skin in an Ag-independent manner. Furthermore, IL-15 administration led to granzyme C upregulation in skin-resident T cells. Thus, the local tissue environment also modulates granzyme C expression in antiviral T cells. These results provide insight into the regulation of expression of granzyme C during antiviral immune responses and suggest a more ubiquitous function for granzyme C than the killing of virus-infected cells.

## Results

### Gamma-delta T cells express granzyme C in the epidermis at steady-state

**Figure 1:**
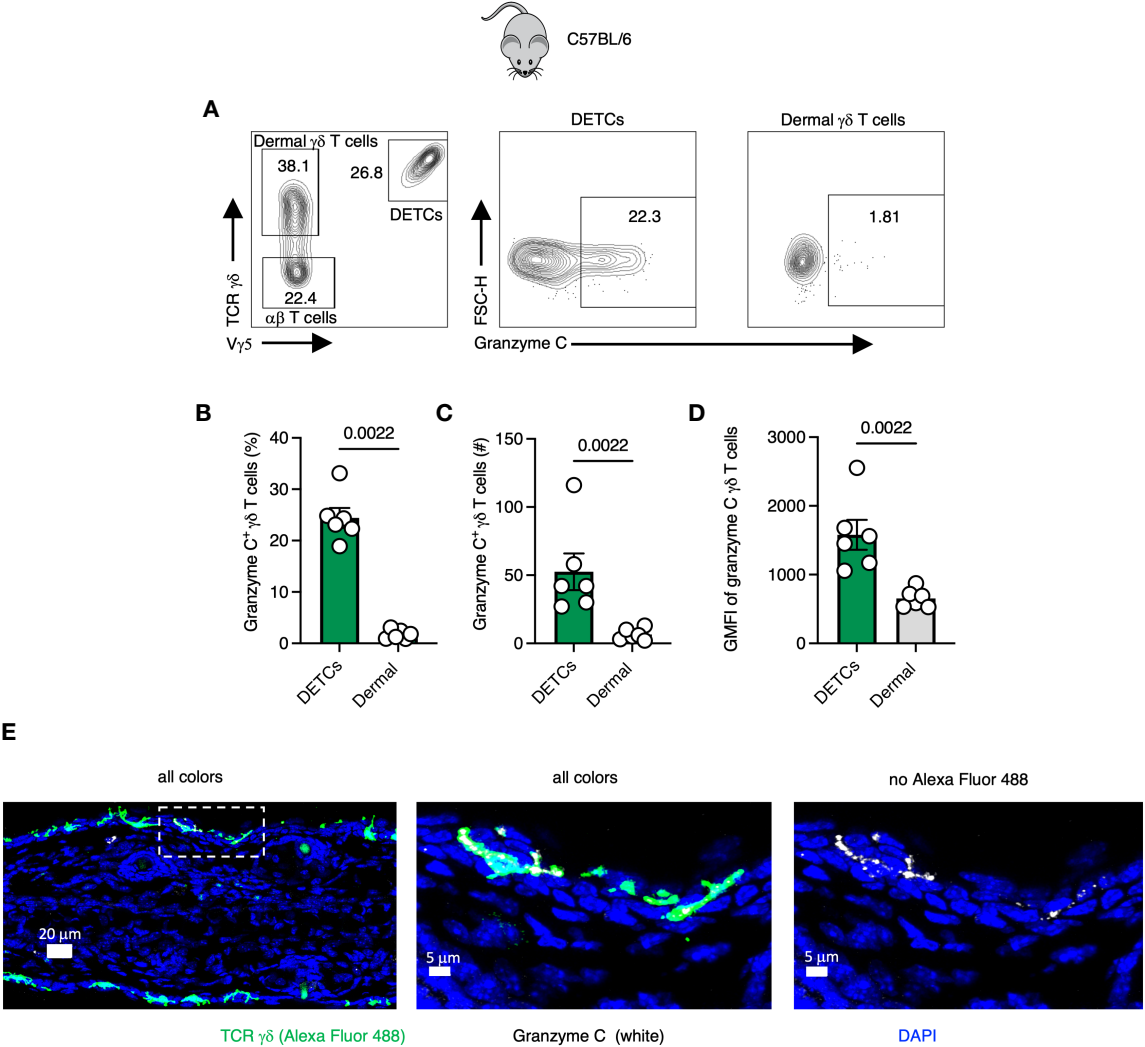
Gamma-delta T cells express granzyme C in the epidermis at steady-state. **(A)** Flow cytometry plots of cutaneous T cells isolated from sex- and age-matched naïve specific-pathogen free C57BL/6 mice. Cells were gated on CD45^+^ CD45.2 IV^-^ CD3^+^. DETCs were gated on CD45^+^ CD45.2 IV^-^ CD3^+^ TCRγδ^+^ Vγ5^+^. Dermal γδ T cells were gated on CD45^+^ CD45.2 IV^-^ CD3^+^ TCRγδ+ Vγ5−. **(B–D)** Percentages, numbers, and mean florescence intensities (MFI) of granzyme C in γδ T cells. Dots represent individual ears. Error bars show the SEM. Results are representative of 3 experiments with 3 mice/group. Statistics = Mann-Whitney tests. **(E)** Confocal images of frozen cross-sections of uninfected ear skin from naïve specific-pathogen free C57BL/6 mice. Boxed area is magnified in panels to the right. Scale bars represent 20 μm (left panel), 5 μm (middle panel), and 5 μm (right panel). Images are representative of at least 3 images taken from 3 mice.

Gamma-delta T cells seed tissues perinatally and can protect against murine poxvirus infection (22, 23). In the skin, Vγ5^+^ dendritic epidermal T cells (DETCs) and Vγ4^+^/Vγ6^+^ γδ T cells occupy the epidermis and dermis, respectively (24, 25). We first analyzed granzyme C expression in both populations using flow cytometry of single-cell suspensions of dissociated skin from wild-type C57BL/6 mice (**Figures 1A–D**). Prior to tissue harvest, we injected a CD45.2-specific antibody (Ab) intravenously (IV) to identify and exclude cells circulating in the vasculature from analysis, as previously described (26). We first gated on CD45.2 IV^-^, CD45^+^, CD3^+^ lymphocytes and used Vγ5 TCR staining to identify TCR γδ^+^ DETCs (with dermal γδ T cells being Vγ5 TCR^−^) (**Figure 1A**). At steady-state in naïve, specific pathogen-free C57BL/6 mice, from 15 to 35% of DETCs expressed granzyme C (**Figure 1B**). Conversely, we did not observe significant frequencies or numbers of granzyme C-expressing dermal γδ T cells (**Figures 1B, C**). Likewise, DETCs expressed significantly higher levels of granzyme C per cell than the few positive dermal γδ T cells, determined by the mean fluorescent intensity (MFI) of intracellular granzyme C staining (**Figure 1D**). As tissue-resident cells can be perturbed by the enzymatic digestion needed to liberate them for flow cytometry (27), we also examined γδ T cells using confocal microscopy of frozen cross-sections of the ear skin (**Figure 1E**). Confocal images corroborated our flow cytometry findings, with a subset of DETCs expressing granzyme C in the epidermis. Interestingly, we often detected granzyme C localized to the dendritic cellular extensions between epidermal keratinocytes (**Figure 1E**). Thus, even naïve, specific-pathogen free mice have granzyme C-expressing skin-resident lymphocytes.

### Gamma-delta T cells increase granzyme C expression during VACV skin infection

Gamma-delta T cells upregulate IFN-γ, granzymes A and B, and perforin during viral infection or in response to PAMPs (28–30). To understand the kinetics of granzyme C expression by DETCs and dermal γδ T cells during VACV infection, we first characterized the γδ T cell response in the skin. We infected C57BL/6 mice with VACV-SIINFEKL (expressing a minigene containing residues 257-264 of ovalbumin) in the ear pinna using a bifurcated needle as previously described (31–33). We used VACV as a viral infection model because this virus infects cells in both the epidermis and dermis where DETCs and dermal γδ T cells reside, respectively (**Figure S1**). We analyzed the single-cell suspensions generated from VACV-infected ears at 0-, 1-, 3-, and 5- days post-infection (dpi) (**Figures S1C–G**). Flow cytometry results revealed that the frequency of DETCs and dermal γδ T cells amongst total leukocytes in the skin decreased significantly at 5 dpi (**Figures S1D–E**). Nevertheless, the total number of DETCs and dermal γδ T cells was significantly higher at 5 dpi (**Figures S1F–G**). Thus, both DETCs and dermal γδ T cells in the skin expand in number during VACV infection but constitute a smaller frequency of CD45^+^ CD3^+^ lymphocytes as new T cells are recruited into the skin.

**Figure 2:**
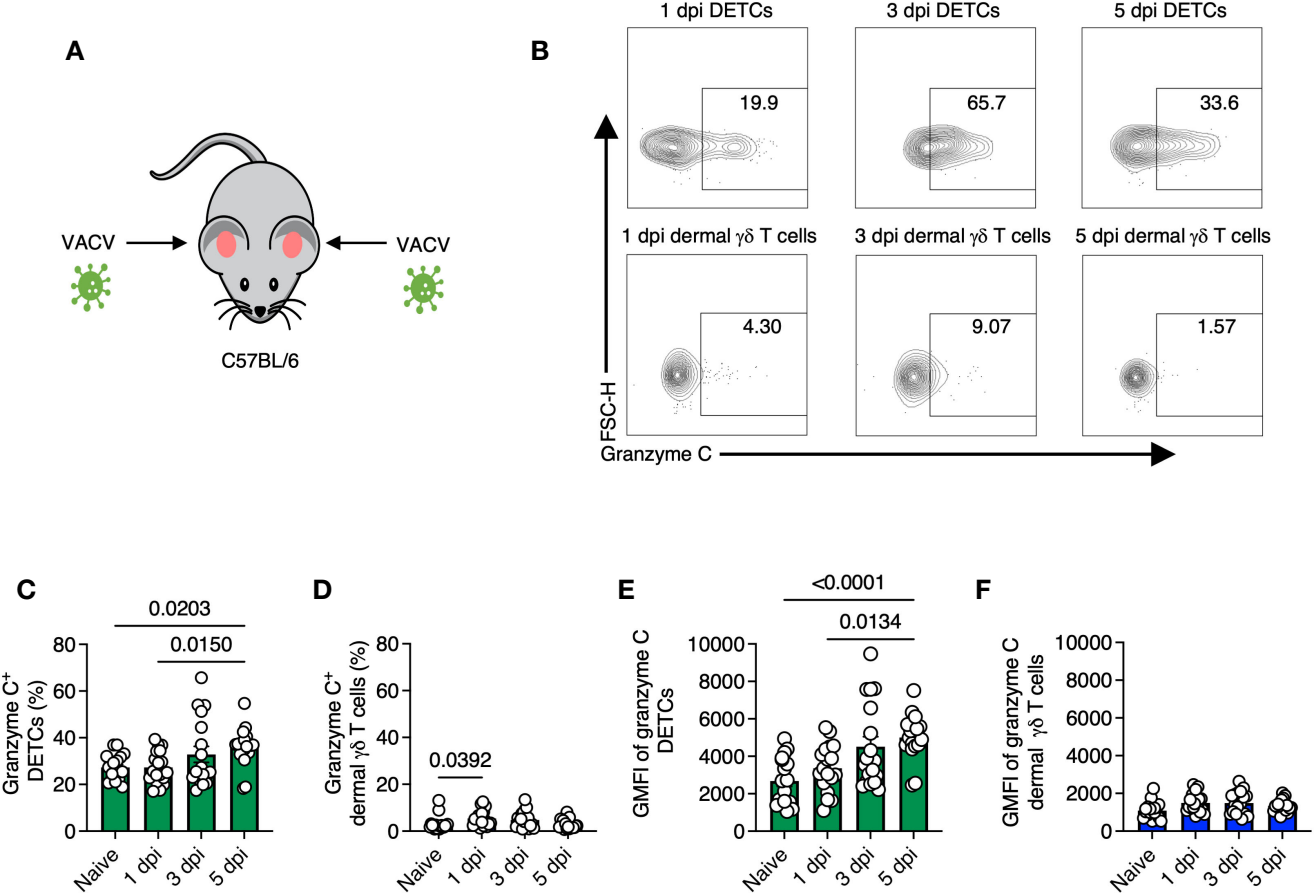
Gamma-delta T cells maintain granzyme C expression during VACV skin infection. **(A)** Diagram of experimental design. Ear pinna of sex- and age-matched C57BL/6 mice were infected epicutaneously with VACV-SIINFEKL. **(B)** Flow cytometry plots of cutaneous γδ T cells isolated from ear pinna of C57BL/6 mice at 1-, 3- 5-dpi with VACV-SIINFEKL. DETCs were gated on CD45^+^ CD45.2 IV^-^ CD3^+^ TCRγδ^+^ Vγ5^+^. Dermal γδ T cells were gated on CD45^+^ CD45.2 IV^-^ CD3^+^ TCRγδ^+^ Vγ5^-^. **(C–F)** Percentages and MFIs of granzyme C in γδ T cells at indicated dpi. MFIs were determined from the entire population (not granzyme C^+^ cells). Dots represent individual ears. Error bars show SEM. Statistics = Kruskal-Wallis tests. Data are pooled from 3 experiments with 3 mice/timepoint.

We next analyzed granzyme C expression in both γδ T cell populations after infection (**Figures 2A–F**). We observed the highest average frequency of granzyme C^+^ DETCs on 5 dpi (~36% compared to ~27% in naïve tissue) (**Figure 2C**). In contrast, the highest frequency of granzyme C^+^ dermal γδ T cells was observed on 1 dpi (5.4 ± 0.81% compared to 3.05 ± 0.71% in naïve tissue) (**Figure 2D**). DETCs expressed more granzyme C per cell than dermal γδ T cells, with the highest granzyme C MFI on 5 dpi (**Figure 2E**). Dermal γδ T cells did not increase granzyme C expression levels by MFI during VACV infection (**Figure 2F**). These data show that epidermal DETCs are the only cutaneous γδ T cell population to upregulate granzyme C during local VACV infection.

We also analyzed the frequencies and numbers of endogenous TCR αβ CD8^+^ T cells that had entered the skin at the same timepoints post-VACV infection (**Figures S2A–F**). At 5 dpi a significant number of CD8^+^ T cells, defined as CD45.2 IV^-^, CD45^+^, CD3^+^, Vγ5^−^, TCR γδ−, CD8β^+^, TCRβ^+^, were recruited to the infected skin (**Figures S2A–C**). Few CD8^+^ T cells expressed granzyme C at this timepoint and the frequency of expression was not increased by infection (**Figures S2D–F**). Together, these data show that epidermal DETCs specifically respond to cutaneous poxvirus infection with enhanced granzyme C production during the first 5 days post-infection.

### Tissue-resident memory CD8^+^ T cells express granzyme C in the skin at steady-state

**Figure 3:**
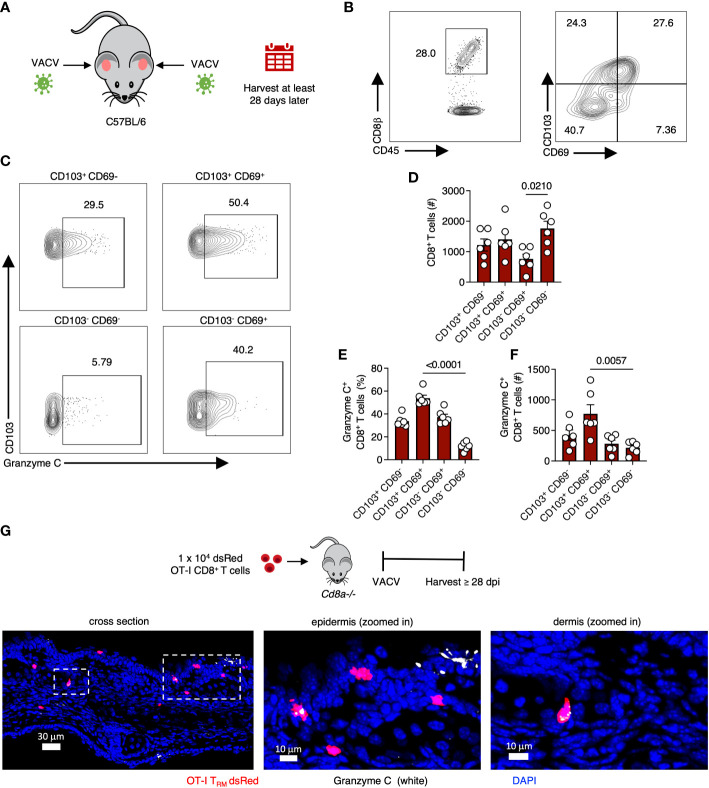
VACV-specific CD8^+^ T cells express granzyme C in the skin. **(A)** Diagram of VACV-SIINFEKL infection model to establish CD8^+^ T_RM_ in the ear pinna of age- and sex- matched C57BL/6 mice. Mice were infected using a bifurcated needle in the ear pinna with VACV-SIINFEKL. T_RM_ were allowed to develop for at least 28 dpi. **(B)** Flow cytometry plots of cutaneous CD8^+^ T cells. Cells were gated on CD45^+^ CD45.2 IV^-^ CD8β^+^. **(C)** Flow cytometry plots showing cutaneous granzyme C^+^ CD8^+^ T cells. CD8^+^ T cells were gated into four quadrants as shown based on the differential expression of CD103 and CD69. **(D)** Total number of CD8^+^ T cells based on differential expression of CD103 and CD69. Dots represent individual ears. Error bars show SEM. Statistics = Kruskal-Wallis tests. Results are representative of 3 independent experiments with 3 mice/group. **(E, F)** As in **(D)** but percentages and numbers of granzyme C^+^ CD8^+^ T cells based on the differential expression of CD103 and CD69. **(G)** Confocal images of frozen cross-sections of ear skin of *Cd8a^−/−^* mice that received 1 x 10^4^ dsRed OT-I CD8^+^ T cells prior to epicutaneous infection with VACV-NP-S-eGFP (containing SIINFEKL). Images were acquired at 28 dpi. Boxed areas are magnified in panels to the right. Scale bars represent 30 μm (left panel), 10 μm (middle panel), and 10 μm (right panel). Images are representative of at least 5 images taken from 2 mice.

We next analyzed CD8^+^ T_RM_ in the skin, another epidermal lymphocyte population with notable antiviral activity (34, 35). We infected mice with VACV-SIINFEKL and allowed for the endogenous polyclonal VACV-specific CD8^+^ T cell response to develop and T_RM_ to form (36). On 28 dpi or greater, we harvested skin, generated single-cell suspensions via enzymatic digestion, and analyzed the frequency of granzyme C expressing T cells using flow cytometry (**Figures 3A–F**). We first gated on CD45^+^, CD45.2 IV^-^, CD8β^+^ cells. Both CD103 and CD69 are commonly used as tissue residency markers to identify T_RM_ in the skin (37). Therefore, we classified CD8^+^ T cells in the skin based on CD103 and CD69 expression and analyzed granzyme C expression in each population (**Figures 3B–F**). CD69^+^ CD103^+^ T_RM_ had the highest frequency of granzyme C expression at approximately 54% of the population. Cells that did not express either CD69 or CD103 had the lowest frequency of granzyme C expression at 11.8 ± 1.5%. We also analyzed granzyme C expression in CD62L^+^ CD44^+^ central memory T cells in the cervical lymph node and spleen. These non-tissue-resident memory CD8^+^ T cells scantly expressed granzyme C (**Figures S3A–C**). Furthermore, circulating CD62L^+^ CD44^-^ naïve, CD62L^+^ CD44^+^ central memory, and CD62L^-^ CD44^+^ effector memory CD8^+^ T cell subsets expressed little granzyme C (**Figures S3D–F**).

As before, we verified granzyme C expression using confocal imaging. For these experiments, we first transferred 1 x 10^4^ dsRed-expressing OT-I TCR transgenic CD8^+^ T cells (recognizing K_b_-SIINFEKL) into *Cd8a^-/-^* mice (deficient in CD8^+^ αβ T cells) to allow easy microscopic visualization of T_RM_ cells in the skin. In contrast to DETCs, we detected OT-I CD8^+^ T cells that expressed granzyme C in both the dermis and epidermis at 28 dpi (**Figure 3G**). We next quantified granzyme C expression in dermal and epidermal OT-I T_RM_ using confocal microscopy (**Figure S4A**). Both dermal and epidermal cells expressed similar levels of granzyme C based on the quantified intensity of granzyme C fluorescence per cell (**Figures S4B–C**).

To complement our confocal and flow cytometric analyses of granzyme C protein expression, we performed single-cell RNAseq on OT-I CD8^+^ T_RM_ isolated from the skin (**Figure S5**). At the mRNA level, approximately 12% of *Cd3e* ^+^ cells also expressed detectable message for granzyme C. OT-I CD8^+^ T cells expressing *Gzmc* also co-expressed *Cd69* and *Itgae* (CD103), consistent with our flow cytometry data. Other additional transcripts that were highly co-expressed with *Gzmc* included *Gzmb, Il2rb, Ifng*, and *Prf1* (perforin).

Together these data show that granzyme C is expressed by resting CD8^+^ T_RM_ in mouse skin.

### OT-I CD8^+^ T_RM_ upregulate granzyme C during secondary VACV infection

**Figure 4:**
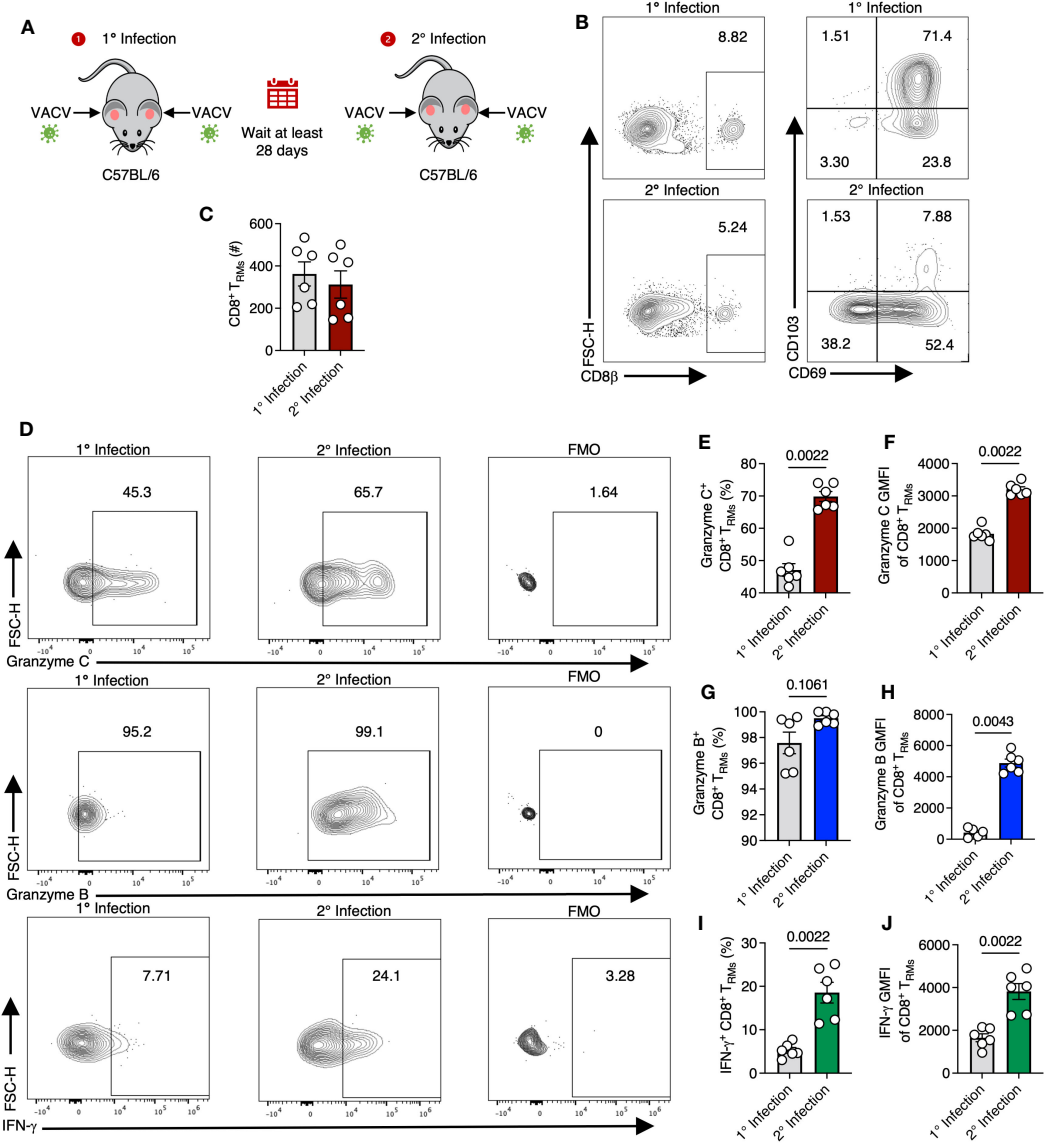
T_RM_ upregulate granzyme C during secondary VACV infection. **(A)** Diagram depicting primary and secondary infection with VACV. C57BL/6 mice were epicutaneously infected in both ears with VACV-SIINFEKL. At least 28 days later, mice were reinfected epicutaneously with the same virus. Ears were removed for analyses on day 2 after secondary infection. **(B)** Flow cytometry plots of cutaneous CD8^+^ T cells after primary or secondary infection with VACV-SIINFEKL. Initial gating was on CD45^+^ CD45.2 IV^-^ cells. **(C)** Total number of CD8^+^ T_RM_ cells gated as CD45^+^ CD45.2 IV^-^ CD8β^+^ CD103^+^ CD69^+^. Dots show individual ears. Error bars show SEM. Statistics = Mann-Whitney test. Results are representative of 1 experiment of 3 with 3 mice/group. **(D)** Flow cytometry plots showing staining for granzyme C (top panels), granzyme B (middle panels), and IFN-γ (bottom panels) in cutaneous CD8^+^ T_RM_ during initial (left panels) or secondary (right panels) VACV infection and corresponding FMOs. **(E–J)** As in **(C)** but percentages and MFIs of CD8^+^ T_RM_ expressing granzymes C, B, and IFN-γ.

We next examined whether VACV-specific T_RM_ would, like DETCs, upregulate granzyme C during VACV infection, or whether this was a specific feature of γδ T cells. For these experiments, we continued analyses of CD103^+^ CD69^+^ VACV-specific CD8^+^ T_RM_ in the skin (37). On 28 dpi or greater, we reinfected the ear pinna of mice with the same VACV that was used for initial infection and analyzed T cells on day 2 post-reinfection (**Figures 4A–C**). Secondary infection increased both the frequency and expression level (MFI) of granzyme C in T_RM_ compared to T_RM_ from mice only infected once (**Figures 4D–F**). Secondary infection also increased the frequency and expression level of granzyme B- and IFN-γ -producing T_RM_ (**Figures 4G–J**). Together, these data demonstrate that CD8^+^ T_RM_ upregulate granzyme C (along with known effector molecules) during re-exposure to VACV.

### OT-I CD8^+^ T_RM_ maintain granzyme C expression in an Ag-independent manner

**Figure 5:**
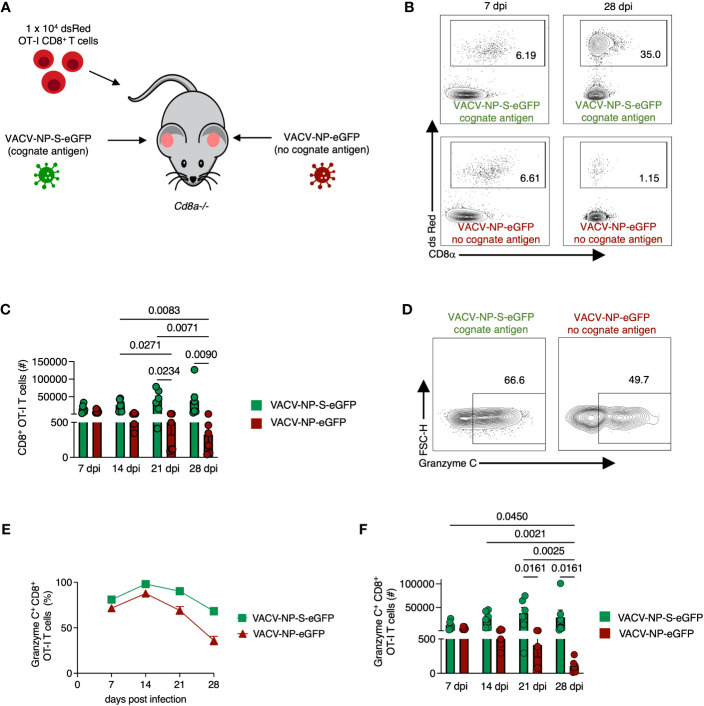
CD8^+^ OT-I T cells recruited to the skin express granzyme C in an antigen-independent manner. **(A)** Experimental design. *Cd8a^−/−^* mice received 1 x 10^4^ dsRed OT-I CD8^+^ T cells prior to epicutaneous infection in one ear with VACV-NP-S-eGFP (expressing the cognate Ag SIINFEKL) and the other ear with VACV-NP-eGFP (no cognate Ag). Ears were harvested at various times post-infection to examine granzyme C expression in ears with or without cognate Ag expression. **(B)** Flow cytometry plots of cutaneous dsRed OT-I CD8^+^ T cells at 7 dpi and 28 dpi isolated from separate ears infected with VACV-NP-S-eGFP (with cognate Ag) or VACV-NP-eGFP (no cognate Ag). Cells initially gated as CD45^+^ CD45.2 IV^-^. **(C)** Numbers of OT-I CD8^+^ T cells present in ears infected with VACV-NP-S-eGFP (with cognate Ag, green bars) or VACV-NP-eGFP (no cognate Ag, red bars) at 7-, 14-, 21-, and 28-dpi. OT-I CD8^+^ T cells were gated as CD45^+^ CD45.2 IV^-^ dsRed^+^ CD8α^+^. Dots represent individual ears. Error bars show SEM. Pooled data are shown from 2 independent timecourse experiments with 3 mice/group. Statistics = Kruskal-Wallis tests. **(D)** Flow cytometry plots of dsRed OT-I CD8^+^ T cells at 28 dpi gated on granzyme C. **(E, F)** As in **(C)** but frequencies and numbers of granzyme C^+^ dsRed OT-I CD8^+^ T cells on the indicated dpi.

Although our data thus far suggested that granzyme C can be upregulated in some antiviral T cells during viral infection, it was unclear whether this was a response to recognition of cognate Ag or inflammation induced by infection. To test whether Ag sensing in the tissue was needed for increased granzyme C expression, we again transferred 1 x 10^4^ naïve OT-I CD8^+^ T cells into *Cd8a* ^-/-^ mice. We then infected one ear with VACV-NP-S-eGFP (expressing a fusion protein consisting of the nucleoprotein from influenza virus, the SIINFEKL OT-I CD8^+^ T cell determinant, and eGFP) and the other ear with VACV-NP-eGFP (an identical virus that lacks SIINFEKL) as previously described (32) (**Figure 5A**). On 7-, 14-, 21-, and 28- dpi, we removed each ear (keeping them separate), created single-cell suspensions, and analyzed cells via flow cytometry (**Figures 5B–F**). The number of OT-I CD8^+^ T cells per ear were similar between ears infected with virus expressing or lacking cognate Ag on days 7 and 14 dpi (**Figure 5C**). On 21 and 28 dpi, we noted a significant increase in the number of OT-I CD8^+^ T cells in the ears infected with VACV-NP-S-eGFP compared to the ears infected with VACV lacking cognate Ag, consistent with previous reports (38) (**Figure 5C**). Granzyme C expression was detectable in OT-I CD8^+^ T cells in the skin by 7 dpi and occurred in T cells present in ears lacking cognate Ag expression (**Figures 5D–F**). Over time, granzyme C expression was maintained in ears lacking cognate Ag expression, although frequencies of granzyme C^+^ OT-I CD8^+^ T cells were slightly higher in ears containing cognate Ag. Accordingly, there were higher numbers of OT-I CD8^+^ T cells expressing granzyme C in the ears containing cognate Ag at 21 and 28 dpi (**Figure 5F**). These data show that CD8^+^ T cells do not require cognate Ag expression in the skin to produce granzyme C; however, cognate Ag may bolster expression.

### OT-I CD8^+^ T_RM_ upregulate granzyme C in response to both viral infection and TCR engagement

**Figure 6:**
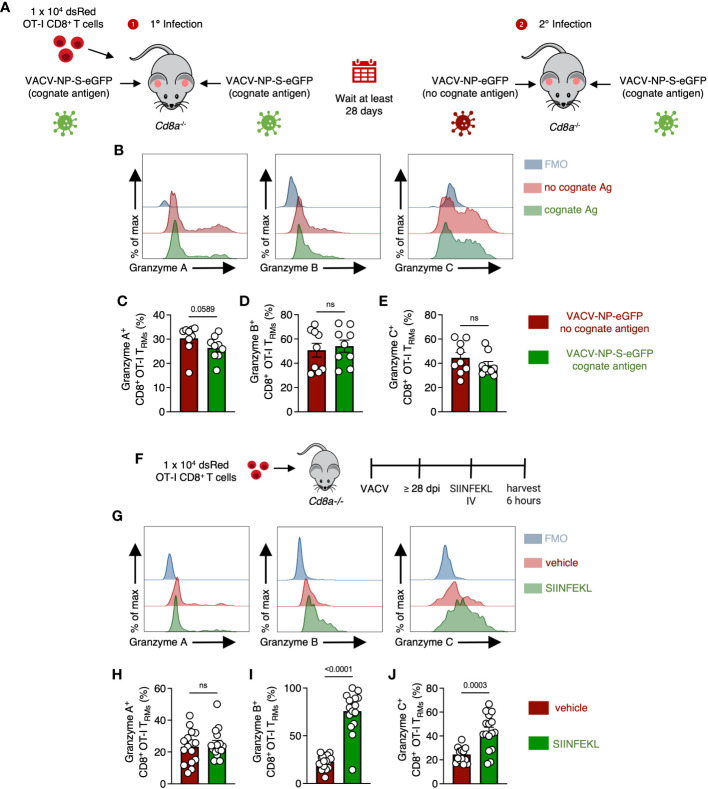
OT-I CD8^+^ T_RM_ can upregulate granzyme C in response to either viral infection or cognate Ag. **(A)** Experimental design. *Cd8a^−/−^* mice received 1 x 10^4^ dsRed OT-I CD8^+^ T cells prior to epicutaneous infection in both ears with VACV-NP-S-eGFP (expressing the cognate Ag SIINFEKL) to establish OT-I CD8^+^ T_RM._ At least 28 days later, mice were infected in one ear with VACV-NP-S-eGFP (expressing the cognate Ag SIINFEKL) and the other ear with VACV-NP-eGFP (no cognate Ag). Ears were removed for analyses on day 2 after secondary infection. **(B)** Overlaid histograms showing expression of granzymes A, B, and C along with corresponding FMOs in OT-I CD8^+^ T_RM_ from ears expressing or lacking cognate antigen. OT-I CD8^+^ T_RMs_ were gated as CD45.2 IV^-^ CD45^+^ CD8β^+^ dsRed^+^ CD69^+^ CD103^+^. **(C–E)** Frequencies of granzyme A^+^ B^+^ or C^+^ OT-I CD8^+^ T_RM_ from ears infected with either VACV-NP-S-eGFP or VACV-NP-eGFP. Dots represent individual ears. Error bars = SEM. Statistics = Mann-Whitney tests. Data are pooled from 2 experiments with 4 or 5 mice/group. **(F)** Experimental design. *Cd8a^−/−^* mice received 1 x 10^4^ dsRed OT-I CD8^+^ T cells prior to epicutaneous infection in both ears with VACV-SIINFEKL. At least 28 days later, mice were IV injected with either SIINFEKL peptide or vehicle control and harvested 6 hours after injection. OT-I CD8^+^ T_RMs_ were gated as CD45.2 IV^-^ CD45^+^ CD8β^+^ dsRed^+^ CD69^+^ CD103^+^. **(G)** As in **(B)** but histograms of granzymes A, B, and C between mice treated with SIINFEKL peptide or vehicle control. **(H–J)** As in **(C–E)** but frequencies of granzyme A^+^, B^+^, or C^+^ OT-I CD8^+^ T_RM_ between mice having received SIINFEKL peptide or vehicle control. Dots represent individual ears. Error bars = SEM. Statistics = Mann-Whitney tests. ns, not statistically significant. Data are pooled from 2 experiments with 4 mice/group.

Although cognate Ag was not needed for continued granzyme C expression, we next queried whether TCR engagement during secondary infection could upregulate granzyme C. As before, we transferred 1 x 10^4^ naïve OT-I CD8^+^ T cells into *Cd8a* ^-/-^ mice and infected both ears with VACV-NP-S-eGFP (containing cognate Ag) to establish OT-I CD8^+^ T_RM_ in both ears under the same conditions. Beyond 28 dpi, we reinfected one ear with VACV-NP-S-eGFP and the other ear with VACV-NP-eGFP (lacking cognate Ag) (**Figure 6A**). Flow cytometric analysis revealed no statistical difference in the frequency of granzyme B and C expressing CD8^+^ T_RM_ in the skin during secondary infection in the presence or absence of cognate Ag (**Figures 6B–E**). Granzyme A expression, however, trended toward increased expression in the absence of cognate Ag (**Figures 6B, C**). Thus, viral infection alone can drive the upregulation of granzyme C in CD8^+^ T_RM_.

In the converse experiment, we assessed whether cognate Ag alone could drive upregulation of granzyme C (without virus-induced inflammation). After the establishment of OT-I CD8^+^ T_RM,_ we injected SIINFEKL peptide intravenously and harvested the ear pinna 6 hours post-injection (**Figure 6F**). Flow cytometric analyses revealed significant upregulation of granzyme C^+^ OT-I CD8^+^ T_RM_ after peptide injection (**Figures 6G–J**). The frequency of granzyme B^+^ T_RM_ also increased even more dramatically, while granzyme A remained relatively unchanged. Together, these data suggest granzyme C can be upregulated by both virally induced inflammation or TCR stimulation in the absence of other inflammatory stimuli. Furthermore, they reveal differential regulation of specific granzyme expression in response to different stimulation.

### Skin-resident T cells upregulate granzyme C in response to IL-15 administration

**Figure 7:**
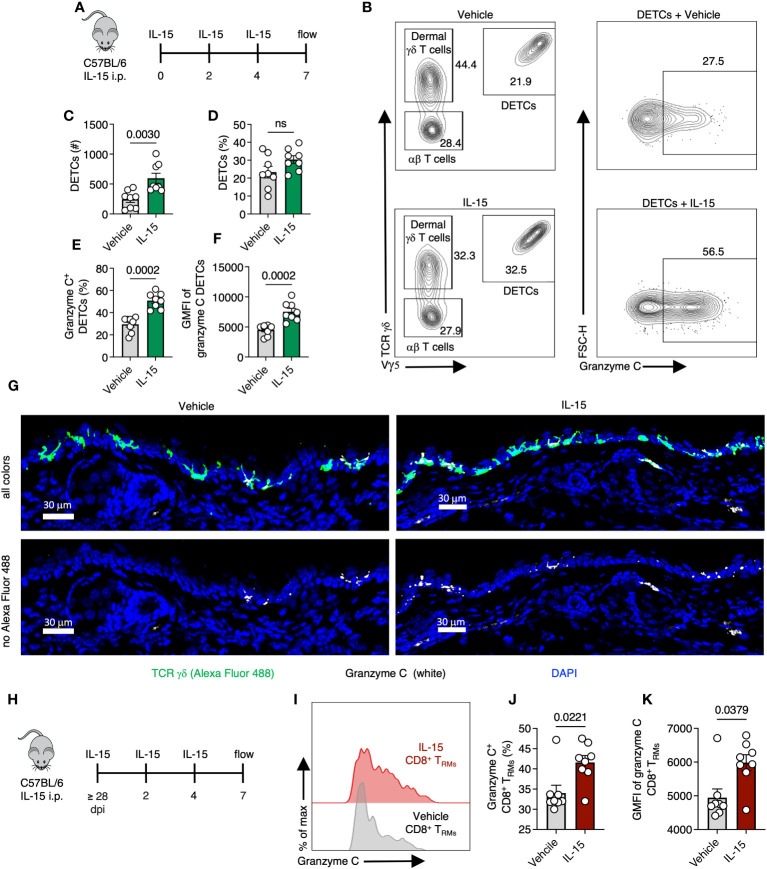
Skin-resident T cells upregulate granzyme C after IL-15 administration. **(A)** Experimental design. Naïve C57BL/6 mice were injected IP with 5 µg of recombinant IL-15 every 48 hr (three treatments). Ear pinna were harvested on day 7 post-treatment. **(B)** Flow cytometry plots of cutaneous T cells from C57BL/6 mice either treated with IL-15 or vehicle control. Cells were gated on CD45^+^ CD45.2 IV^-^ CD3^+^. DETCs were gated on CD45^+^ CD45.2 IV^-^ CD3^+^ TCRγδ^+^ Vγ5^+^. **(C, D)** Numbers and frequencies of DETCs isolated C57BL/6 mice either treated with IL-15 or vehicle control. Dots represent individual ears. Error bars = SEM. Statistics = Mann-Whitney tests. ns, not statistically significant. Data representative of 2 independent experiments with 4 mice/group. **(E, F)** As in **(C, D)** but frequencies of granzyme C^+^ DETCs and MFIs of granzyme C in all DETCs in IL-15- or vehicle-treated mice. **(G)** Confocal images of frozen cross-sections of ears of naive C57BL/6 mice either treated with IL-15 or vehicle control. Top images show all colors green (TCR γδ AlexFluor 488), white (granzyme C), blue (DAPI-nuclear stain). Bottom images remove green (TCR γδ AlexFluor 488) channel to better reveal granzyme C signal (white). Scale bars represent 30 μm. Images are representative of at least 3 images taken from 3 mice/group. **(H)** Experimental design. Age- and sex- matched C57BL/6 mice were infected with VACV-SIINFEKL for at least 28 days for T_RM_ formation. At least 28 days post-infection, mice were injected IP with IL-15 every 48 hr. Ear pinna were harvested on day 7 post-treatment. **(I)** Overlaid histograms of granzyme C expression in CD8^+^ T_RM_ in IL-15- or vehicle-treated mice. CD8^+^ T_RM_ gated on CD45.2 IV^-^, CD45^+^, CD3^+^, Vγ5^−^, TCR γδ^−^, CD8β^+^, CD103^+^, CD69^+^ cells. **(J, K)** Frequencies of granzyme C^+^ CD8^+^ T_RM_ and granzyme C MFIs in all T_RM_ between treatment groups. Dots represent individual ears. Error bars = SEM. Statistics = Mann-Whitney tests. Data representative of 2 independent experiments with 4 mice/group.

We next explored whether local cytokine changes could promote granzyme C expression in the absence of cognate Ag recognition. The cytokine IL-15 is induced during many acute viral infections and is important for tissue-resident lymphocyte maintenance (39, 40). *In vitro*, IL-15 upregulates granzyme C expression in isolated liver ILC1s (13). We therefore explored whether *in vivo* treatment with IL-15 alone could induce granzyme C expression. We first assessed the effect of IL-15 administration on DETCs. We intraperitoneally (IP) injected naïve wild-type C57BL/6 mice with exogenous IL-15 every 48 hr (three treatments) and harvested the ear pinna on day 7 (**Figure 7A**). Mice from the IL-15 treatment group had a higher number but not frequency of DETCs compared to the vehicle control (**Figures 7B–D**). DETCs in mice treated with IL-15 had an increased frequency of granzyme C expression and MFI compared to DETCs in mice receiving vehicle control (**Figures 7E, F**). IL-15 treatment also increased the expression level (MFI) and frequency of dermal γδ T cells expressing granzyme C, though this remained low compared to DETCs (**Figure S6**). Confocal imaging also revealed numerous granzyme C^+^ DETCs in IL-15-treated mice (**Figure 7G**).

Having demonstrated exogenous IL-15 administration could upregulate granzyme C expression in skin-resident γδ T cells, we examined whether IL-15 would have the same effect in CD103^+^ CD69^+^ CD8^+^ T_RM_. As before, we infected C57BL/6 mice with VACV-SIINFEKL and allowed for the endogenous polyclonal VACV-specific CD8^+^ T cell response to develop (36). On 28 dpi or greater, we administered IL-15 via IP injection as before, generated single-cell suspensions via enzymatic digestion from the ear pinna and analyzed the frequency of granzyme C-expressing T cells using flow cytometry (**Figures 7H–K**). Like the DETCs, IL-15 increased T_RM_ expression of granzyme C (in both frequency and MFI) compared to vehicle controls (**Figures 7I–K**). Thus, granzyme C expression is also cytokine responsive *in vivo*.

## Discussion

Mice can express many different granzymes, some of which do not have established function or known immunological roles. One of these, granzyme C was recently identified as a definitive marker for mature antiviral ILC1s as these cells continually produce granzyme C in the liver (13). However, it was unknown if other antiviral lymphocytes express granzyme C and whether its expression is regulated *in vivo* by viral infection. Here, using flow cytometry, confocal microscopy, and single-cell RNA-seq, we demonstrate that different innate and adaptive antiviral T cell subsets express granzyme C in the skin. During homeostasis, some DETCs and most T_RM_ present in the epidermis expressed granzyme C. Poxvirus infection of the skin upregulated granzyme C production by both DETCs and T_RM_. Interestingly, cognate Ag recognition in the tissue was not required for maintained granzyme C expression by TCR-transgenic OT-I CD8^+^ T cells. Nonetheless, cognate Ag recognition enhanced granzyme C expression. Additionally, IL-15 treatment also enhanced granzyme C expression by DETCs and virus-specific T_RM_. Together, our data reveal that granzyme C expression is more widespread than previously appreciated and is responsive to both environmental cues and TCR engagement.

An important question remains: what is the function of homeostatic granzyme C expression? Other studies have shown that granzyme C can be expressed without contact with tumor or virally infected cells, hinting at other roles for granzyme C besides the direct cytolysis of target cells. Most experiments examining granzyme C-mediated cytolysis have been performed *in vitro* (3, 11, 13, 41, 42). The most compelling *in vivo* data for granzyme C-mediated killing demonstrated that the constitutive activation of granzyme C^+^ cells led to perforin-dependent lethality in uninfected neonatal mice (13). However, perforin knockout mice also succumbed to death in this model, albeit with a delay of several weeks. Furthermore, the crystal structure of granzyme C has revealed that this protease may be auto-inhibited under normal circumstances (43). Thus, the function of granzyme C may be multifactorial but remains unestablished.

There are demonstrated non-canonical roles for other granzymes. Some have been shown to play a pro-inflammatory role during infection. Granzymes A and B in humans and mice are produced at high levels during various viral infections including HIV, CHIKV, and EBV (6, 44, 45). In a mouse model of CHIKV infection, granzyme A promoted arthritic foot swelling but not viral clearance (45). Granzyme K is a marker of a unique age-associated CD8^+^ T cell population in both humans and mice, which may induce fibroblast secretion the pro-inflammatory cytokines IL-6, CCL2, and CXCL1 (7). Granzyme C can be upregulated in mast cells activated with IL-33 (46).

Granzymes can also directly inhibit viruses via the cleavage of viral proteins (6). Murine granzyme B degrades the herpes simplex virus type 1 (HSV-1) immediate early protein ICP4 (needed for transcription of early and late viral genes) (47, 48). Granzyme M inhibits human cytomegalovirus (HCMV) replication through the cleavage of the viral protein pp71 (49). Additionally, granzyme H, the human ortholog of granzyme C, can cleave DNA-binding protein and the granzyme B-inhibiting 100k assembly protein of adenovirus (50). Future studies will be needed to determine whether granzyme C also has direct antiviral effects through the degradation of specific viral proteins.

IL-15 is an important cytokine for tissue-resident antiviral protection. DETCs, T_RM_, and ILC1s all reside in barrier epithelia, and all require IL-15 for their development and/or maintenance (25, 51–53). Originally identified as a T cell proliferation factor, IL-15 can be expressed throughout the body by antigen-presenting cells (APCs), bone-marrow stromal cells, and epithelial lineages such as human and mouse epidermal keratinocytes (54–57). IL-15 is expressed as both soluble and trans-presented forms, the latter of which is thought to represent the most physiologically relevant form of the cytokine (58). During IL-15 trans-presentation, IL-15 is bound to the IL-15 receptor α (IL-15Rα) and traffics to the cell surface as an IL-15/IL-15Rα complex (58). Stimulation by IL-15 therefore requires contact between the recipient cells and IL-15/IL-15Rα trans-presenting cell (59). We show here that IL-15 upregulates granzyme C expression in tissue-resident lymphocytes *in vivo*. IL-15 may enhance granzyme C expression independent from its canonical function, for example as a consequence of cytokine-driven cellular expansion. Alternatively, granzyme C, a serine protease, may help to liberate this or other cytokines for use by tissue-resident lymphocytes. The unique positioning of DETCs within the epidermis and the expression of granzyme C along their dendritic extensions of DETCs might also suggest a strategy to distribute granzyme C as widely as possible in the epidermis.

Granzyme C upregulation after IL-15 stimulation suggests that granzyme C expression might serve as a surrogate to identify cells receiving IL-15 during homeostasis, infection, and inflammation. Interestingly, human granzyme K expression was identified as a feature of both γδ T cell and innate CD8^+^ T cell subsets and is upregulated in response to cytokine stimulation rather than TCR stimulation (60). Our data reveal that a smaller percentage of DETCs express granzyme C than CD8^+^ T_RM_, at least at the timepoints we examined. This may reflect the recent development of T_RM_ in the epidermis and more recent IL-15 acquisition by T_RM._ Interestingly, T_RM_ and DETCs exhibit different motility in the epidermis (35), which might cause differences in IL-15 acquisition as these cells perambulate through the keratinocytes.

Our data provide a framework for understanding granzyme C expression by antiviral lymphocytes in the skin. Rather than being developmentally programmed, granzyme C expression was dynamic and reflected the current tissue status. Given the functional versatility of granzymes, the deletion of granzyme C in select lymphocyte populations may disrupt viral clearance either through reduced lysis of virally infected cells or the inhibition/degradation of viral proteins. Alternatively, knocking out granzyme C may impair pro-inflammatory pathways or reduce the ability of lymphocytes to migrate through the dense tissue microenvironment. Although CRISPR editing is an attractive approach to knockout granzyme C expression in OT-I CD8^+^ T_RM_, granzyme gene homology will necessitate careful validation to ensure proper targeting of only granzyme C. Furthermore, this approach could not be employed for other tissue-resident cells that are seeded embryonically or neonatally. Therefore, the creation of animal models deleting granzyme C expression will be required to fully unravel the role(s) of this enigmatic protease during antiviral immune responses. Nonetheless, the widespread increase in granzyme C expression in skin-resident lymphocytes in response to viral infection or cytokine stimulation suggests that this protease, like other granzymes, could be an important contributor to antiviral immunity in the tissue.

## Materials and methods

### Mice

Specific pathogen-free C57BL/6N mice were obtained from Taconic Farms. dsRed (Stock Tg(CAG-DsRed*MST)1Nagy/J, #5441); *Cd8a^-/-^ (B6.129S2-Cd8a^tm1Mak^/J*, #2665*)*; and albino C57BL/6 (B6(Cg)-*Tyr^c-2J^*/J, #58) mice were obtained from Jackson Laboratories. *Rag1^-/-^* (Line 146); *Rag2^-/-^Il2rg^-/-^* (Line 111); *T-betZsGreen* (Line 8419); and OT-I TCR transgenic (C57BL/6NAi-[Tg]TCR OT-1-[KO]RAG1, Line 175) mice were obtained from the NIAID Intramural Research Repository at Taconic Farms. dsRed mice were crossed with OT-I TCR transgenic mice to create dsRed OT-I mice. *Rag1^-/-^* mice were crossed with *T-betZsGreen* mice and bred to homozygosity to create *Rag1^-/-^ T-betZsGreen* mice. *Cd8a^-/-^* mice were crossed with B6 albino mice and bred to homozygosity to create albino *Cd8a^-/-^* mice. 6- to 20-week-old male and female mice were used in experiments. All mice were maintained on standard rodent chow and water supplied as necessary. All animal studies were approved by and performed in accordance with the Animal Care and Use Committee of NIAID.

### Microbe strains

Viruses used for this study included VACV-NP-S-eGFP (expressing a fusion protein consisting of influenza nucleoprotein, the SIINFEKL T cell determinant, and eGFP); VACV-NP-eGFP (an identical virus to VACV-NP-S-eGFP that lacks SIINFEKL); VACV-SIINFEKL (expressing residues 257-264 of ovalbumin). Recombinant VACV viruses were generated as TK^-^ viruses using the Western Reserve strain of VACV and have been previously described (32, 61).

### Method details

#### Viral infections and enzymatic tissue dissociation

Mice were infected in the dorsal ear pinna as previously described (33, 62) with 5 pokes of a bifurcated needle dipped in VACV. VACV infection was performed with VACV-SIINFEKL (1 x 10^8^ pfu), VACV-NP-S-eGFP (2.1 x 10^8^ pfu), or VACV-NP-eGFP (2.4 x 10^8^ pfu). At the indicated time, ears were harvested, separated into dorsal and ventral sides, diced, and digested in RPMI containing 7.5% fetal bovine serum (FBS), collagenase I (Worthington), DNAse (Worthington), and brefeldin A solution 1000x (Biolegend) at a 1:1000 dilution for 1 hr at 37°C. Spleens and cervical lymph nodes were harvested and homogenized using a pestle in RPMI containing 7.5% FBS and brefeldin A solution 1000x (Biolegend) at a 1:1000 dilution.

### Blood collection and lymphocyte isolation

Prior to blood collection mice were IV injected with 200 µls of saline containing brefeldin A solution (Biolegend) at a 1:100 dilution. Mice were immediately placed under isoflurane anesthesia and blood was collected through terminal retro-orbital eye bleeds. BioWhittaker Lymphocyte Separation Medium (LSM) (Lonza) was then used to isolate lymphocytes.

### Flow cytometry analyses

To distinguish IV^+^ cells, mice were injected with 3 µgs of pacific blue-conjugated CD45.2 (clone 104.2) intravenously as previously described 3 minutes prior to tissue isolation (63). Suspensions were filtered through 70 µm nylon cell strainers. Cells were stained with a combination of the following antibodies: CD45 (clone 30-F11), CD45.2 (clone 104), CD3 (clone 17A2), CD8β (clone H35-17.2 or YTS156.7.7), CD8α (clone 53-6.7), CD69 (clone H1.2F3), CD103 (clone 2E7), Vα2 (clone B20.1), TCRβ (clone H57-597), TCR γδ (clone GL3), Vγ5 (Tonegawa’s nomenclature (clone 536)), granzyme A (GzA-3G8.5), granzyme B (clone 16G6 or NGZB), granzyme C (clone SFC1D8), IFN-γ (clone XMG1.2), Armenian Hamster IgG Isotype Control (clone HTK888), CD62L (clone MEL-14), CD44 (clone IM7) and fixable viability dyes (Zombie Aqua) from Biolegend, eBiosciences, Invitrogen, or BD Biosciences diluted in PBS and brefeldin A solution (Biolegend) at a 1:1000 dilution. Cells were fixed with 3.2% paraformaldehyde for 15 minutes and intracellular staining was done using 0.5% saponin in Hanks Balanced Salt Solution (HBSS) + 0.1% Bovine Serum Albumin (BSA) for 1 hr at room temperature. dsRed^+^ cells were identified based upon fluorescent protein expression. Cells were analyzed on a Fortessa flow cytometer (BD Biosciences) or 5L 16UV-16V-14B-10YG-8R Aurora (Cytek) and resultant data analyzed using FlowJo software (Treestar).

### Drug and Peptide treatment

Recombinant murine IL-15 (PeproTech) was reconstituted in water and diluted in sterile saline solution prior to intraperitoneal (IP) injection of 5 µg/mouse every 48 hr for a total of 3 treatments. Ears were harvested 7 days after the start of the first treatment. SIINFEKL peptide (GenScript) was reconstituted in dimethyl sulfoxide (DMSO) and diluted in sterile saline solution prior to one time IV injection of 200 µg of peptide/mouse. Ears were harvested 6 hours after peptide injection.

### Adoptive transfer of OT-I CD8^+^ T cells

CD8^+^ T cells were purified from spleens and lymph nodes using an EasySep Mouse CD8^+^ T cell Isolation Kit (Negative Selection) according to the manufacturer’s instructions (Stem Cell Technologies). Cells were naïve (CD69^-^) (antibody information clone: H1.2F3) and > 90% pure by flow cytometry prior to IV transfer. Unless otherwise noted, mice received a standard dose of 1 x 10^4^ OT-I CD8^+^ T cells prior to infection.

### Confocal microscopy of frozen tissue sections

Ears were removed on the indicated dpi, fixed in periodate-lysine-paraformaldehyde (PLP) for 24 hr, and moved to 30% sucrose/PBS solution for 24 hr. Ears were embedded in optimal-cutting-temperature (OCT) medium (Electron Microscopy Sciences) in cross-section orientation and frozen in dry-ice-cooled 2-methylbutane. 16-µm sections were cut on a Leica cryostat (Leica Microsystems), blocked with HBSS, 0.1% BSA, 10% bovine and donkey serum, 0.05% Triton X. Tissues were stained with a combination of the following antibodies: purified granzyme C (clone SFC1D8), Alexa Fluor 647 AffiniPure Goat Anti-Armenian Hamster IgG (H+L) or Alexa Fluor^®^ 594 AffiniPure Goat Anti-Armenian Hamster IgG (H+L), purified Cytokeratin 6 (clone SP87), Alexa Fluor 647 AffiniPure Donkey Anti-Rabit IgG (H+L), conjugated Alexa Fluor 488 anti-mouse TCR γ/δ (clone GL3) antibody, and nuclei stained using DAPI from Biolegend, ThermoFisher, or Jackson ImmunoResearch. Antibodies were diluted in HBSS, 0.1% BSA, 10% bovine and donkey serum, 0.05% Triton X. Images were acquired on a Leica SP8 confocal microscope equipped with hybrid detectors or a Leica Stellaris 8.

### Cell sorting and single-cell RNA-seq

*Rag1^-/-^ T-betZsGreen* mice received 1 x 10^4^ dsRed OT-I CD8^+^ T cells prior to infection with VACV-SIINFEKL. At the indicated time post infection, mice were injected with 3 µgs of pacific blue-conjugated CD45.2 (clone 104.2) IV as previously described 3 minutes prior to tissue isolation to distinguish IV^+^ cells (63). Ears were harvested, separated into dorsal and ventral sides, diced, and digested in RPMI containing 7.5% fetal bovine serum (FBS), collagenase I (Worthington), and DNAse (Worthington) for 1 hr at 37°C. Suspensions were filtered through 70 µm nylon cell strainers. Cells were stained with a combination of the following antibodies: CD45 (clone 30-F11), CD8α (clone 53-6.7) along with Zombie Aqua viability dye. Using a BD FACS Aria III Cell Sorter, OT-I CD8^+^ T cells and Tbet-ZsGreen group I ILCs were sorted as Viability Dye^-^ CD45.2 IV^-^ CD45^+^ dsRed^+^ CD8α^+^ or ZsGreen^+^ cells, respectively, into RPMI containing 10% FBS and HEPES (25 mM). Cells were centrifuged and resuspended in RPMI containing 10% FBS and HEPES (25 mM) at a 1000 cells/μl concentration.

### Single-cell RNA-seq library generation

Two technical replicates of the *Rag1^-/-^ T-betZsGreen* ear samples were collected for single-cell RNA sequencing using Chromium Next GEM Single Cell 3’ v3.1 standard kit (10X Genomics, Pleasanton, CA). Approximately 3000 cells were targeted in each single-cell preparation. For the preparation of the cDNA and sequencing library generation, libraries were prepared as described in the Chromium Next GEM Single Cell 3’ Reagent Kit v3.1 (Dual Index) user guide to produce barcoded cDNA and perform Illumina sequencing library preparation.

### Single-cell RNA-seq library sequencing, and analysis

The Illumina library quality was assessed by TapeStation D1000 high sensitivity reagent kit (Agilent, Santa Clara, CA), and DNA concentration was measured by Qubit High Sensitivity reagent kit (Thermo Fisher, Waltham, MA). Samples were diluted for sequencing and pooled equimolarly according to Illumina sequencing protocol to a final concentration of approximately 650 pM. Sequencing was performed NextSeq2000 instrument using two P3 200 cycle kits (Illumina, San Diego, CA) to target approximately 50,000 reads per cell.

After sequencing, the FastQ files were submitted to Cell Ranger ‘count’ and ‘aggregate’ functions. Sample *Rag1^-/-^ T-betZsGreen* Ear_1 had 2,744 passing cells with 1,986 median genes per cell and *Rag1^-/-^ T-betZsGreen* Ear _2 had 1,910 passing cells with 2,180 median genes per cell. Sequencing saturation for both libraries were 64.8% and 76.8%, respectively. After all quality control of the replicates, the expression of granzyme C and other genes were analyzed in the *Loupe* browser (10X Genomics, Pleasanton, CA).

### Statistical analyses

Significances were assessed using Prism software (GraphPad) using a Kruskal-Wallis Test (3 or more groups) or unpaired two-tail Mann-Whitney test (2 groups) as indicated in the figure legends. Exact P values are shown throughout, statistical significance was set at P ≤ 0.05.

## Data availability statement

The raw data supporting the conclusions of this article will be made available by the authors, without undue reservation.

## Ethics statement

The animal study was approved by the Animal Care and Use Committee of NIAID, NIH. The study was conducted in accordance with the local legislation and institutional requirements.

## Author contributions

RL and HH conceived and designed the study. RL, PD, and HH analyzed the data. RL and HH drafted the manuscript. RL, LP, JS, and ND performed experiments. HH provided reagents, experimental expertise, and reviewed the manuscript. All authors contributed to the article and approved the submitted version.
