# Supplementary material for: Widespread and dynamic expression of granzyme C by skin-resident antiviral T cells
Source: Front Immunol. 2023 Sep 21;14:1236595. doi: 10.3389/fimmu.2023.1236595 (PMC10552530; doi:10.3389/fimmu.2023.1236595)
Supplement: Supplementary file 1 [file DataSheet_1.pdf]

Supplementary Material

Widespread and dynamic expression of Granzyme C by skin-resident antiviral T cells

Ramon A. Lujan, Luxin Pei, John P. Shannon, Nathânia Dábilla, Patrick T. Dolan, and Heather D. Hickman*

*** Correspondence:** Heather D. Hickman: hhickman@mail.nih.gov*

Figure S1.

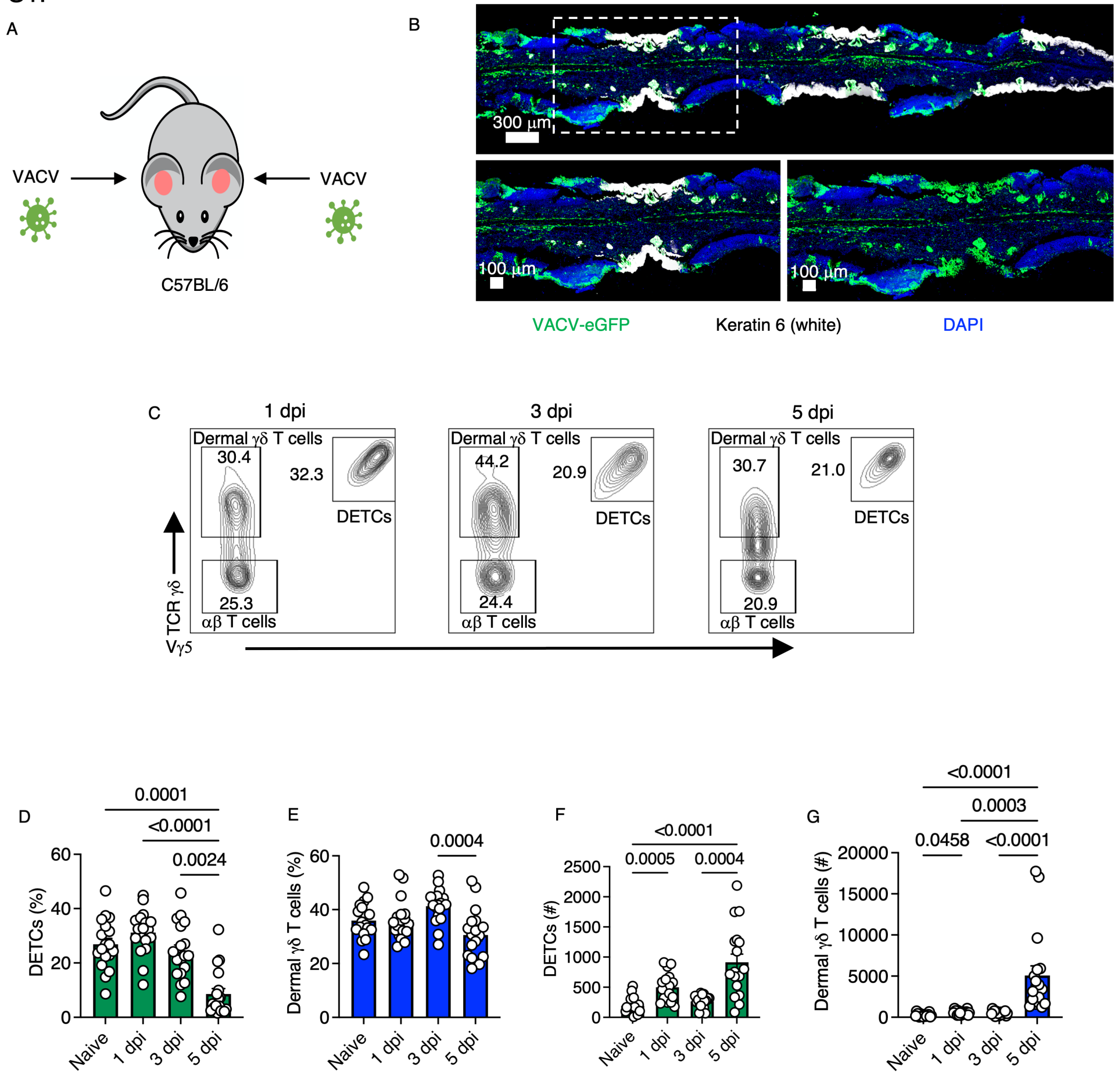

Supplementary Figure 1. Kinetics of γδ T cells during VACV skin infection.

(**A**) Diagram of experimental design. Ear pinna of sex- and age-matched C57BL/6 mice were infected epicutaneously with VACV-SIINFEKL. **(B**) Confocal images of a *Rag2*$^{-/-}$*Il2rg*$^{-/-}$ mouse that received 2.5 x 10^5 OT-I $CD8^+$ T cells prior to infection with VACV-NP-eGFP (expressing GFP from a viral promoter). Skin was harvested on day 10 pi. Frozen cross sections were stained with keratin 6 (white) to reveal areas of keratinocyte proliferation. Green=VACV-infected cells; white=keratin 6; blue=DAPI. Boxed area is magnified in panels on the bottom. Scale bars represent 300 µm (top panel), 100 µm (lower left panel), and 100 µm (lower right panel). Image representative of at least three images taken from two mice. (**C**) Contour plots generated by flow cytometry showing cutaneous γδ T cells isolated from sex- and age-matched C57BL/6 mice at 1-, 3-, 5-dpi with VACV-SIINFEKL. Cells were first gated on $CD45^+$ CD45.2 IV^- $CD3^+$. (**D-G**) Percentages (D-E) and numbers (F-G) of cutaneous γδ T cells at indicated day pi. with VACV-SIINFEKL in C57BL/6 mice.

DETCs were gated on CD45+ CD45.2 IV- CD3+ TCRγδ+ Vγ5+. Dermal γδ T cells were gated on CD45+ CD45.2 IV- CD3+ TCRγδ+ Vγ5-. Dots represent individual ears. The complete time course was performed 3 times with 3 mice/group. Data were pooled from 3 experiments. Error bars = SEM. Statistics = Kruskal-Wallis tests.

Figure S2.

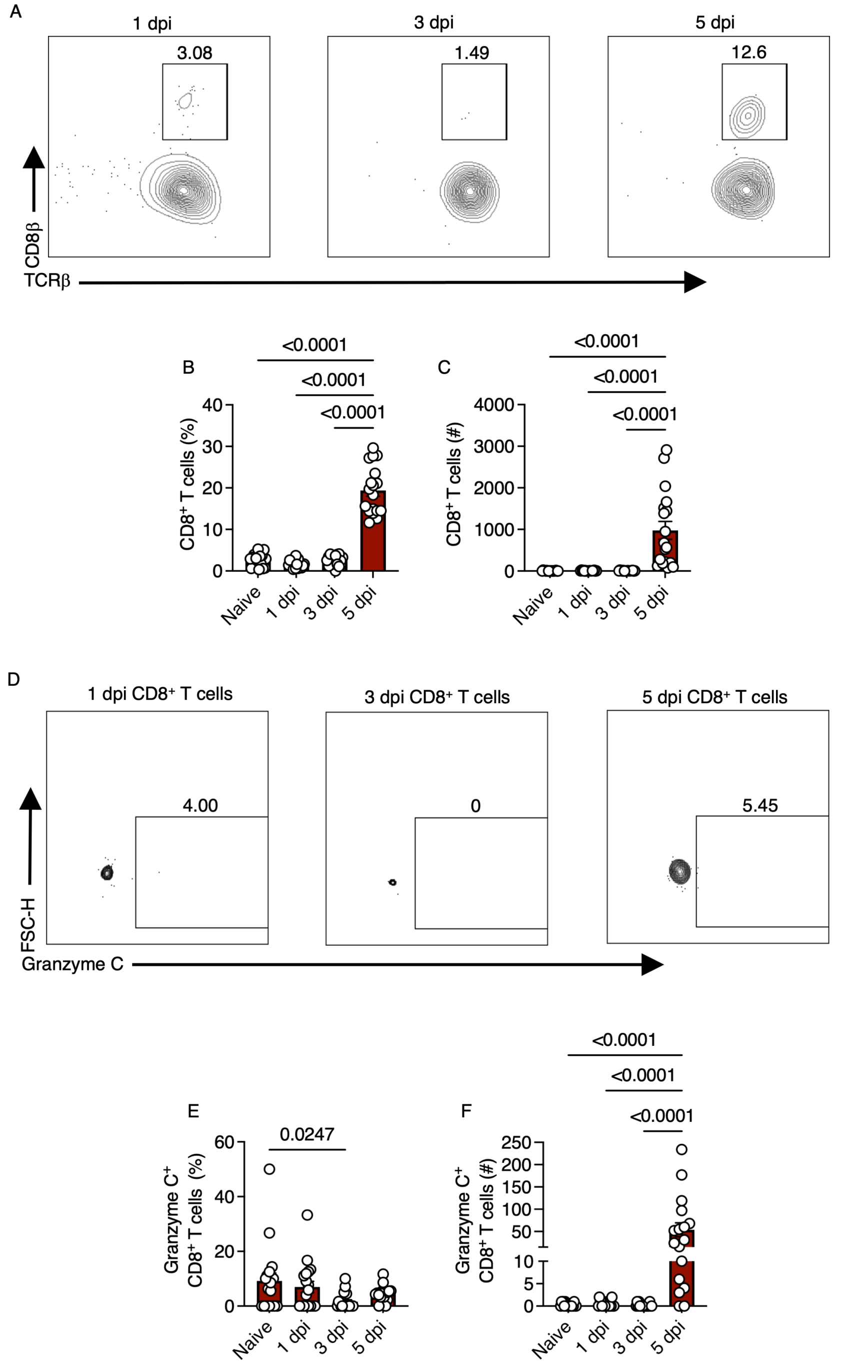

Supplementary Figure 2. CD8+ T cell kinetics during early VACV infection.

(**A**) Flow cytometry plots showing cutaneous T cells isolated from sex- and age-matched C57BL/6 mice at 1-, 3-, 5- dpi with VACV-SIINFEKL. Cells were first gated on CD45.2 IV^- $CD45^+$ $CD3^+$ $V\gamma5^-$ TCR $\gamma\delta^-$. (**B-C**) Percentages (B) and numbers (C) of $CD8^+$ T cells throughout the first 5 days of infection. Cells were gated on CD45.2 IV^- $CD45^+$ $CD3^+$ $V\gamma5^-$ TCR $\gamma\delta^-$ $CD8\beta^+$ $TCR\beta^+$. Dots represent individual ears. Error bars = SEM. Statistics = Kruskal-Wallis tests. (**D**) Flow cytometry plots of granzyme C^+ $CD8^+$ T cells isolated from C57BL/6 mice at 1-, 3- 5-dpi with VACV-SIINFEKL. (**E-F**) Percentages (E) and numbers (F) of granzyme C^+ $CD8^+$ T cells throughout the first 5 days of infection. The complete time course was performed 3 times with 3 mice/group; data were pooled. Dots represent individual ears. Error bars = SEM. Statistics = Kruskal-Wallis tests.

Figure S3.

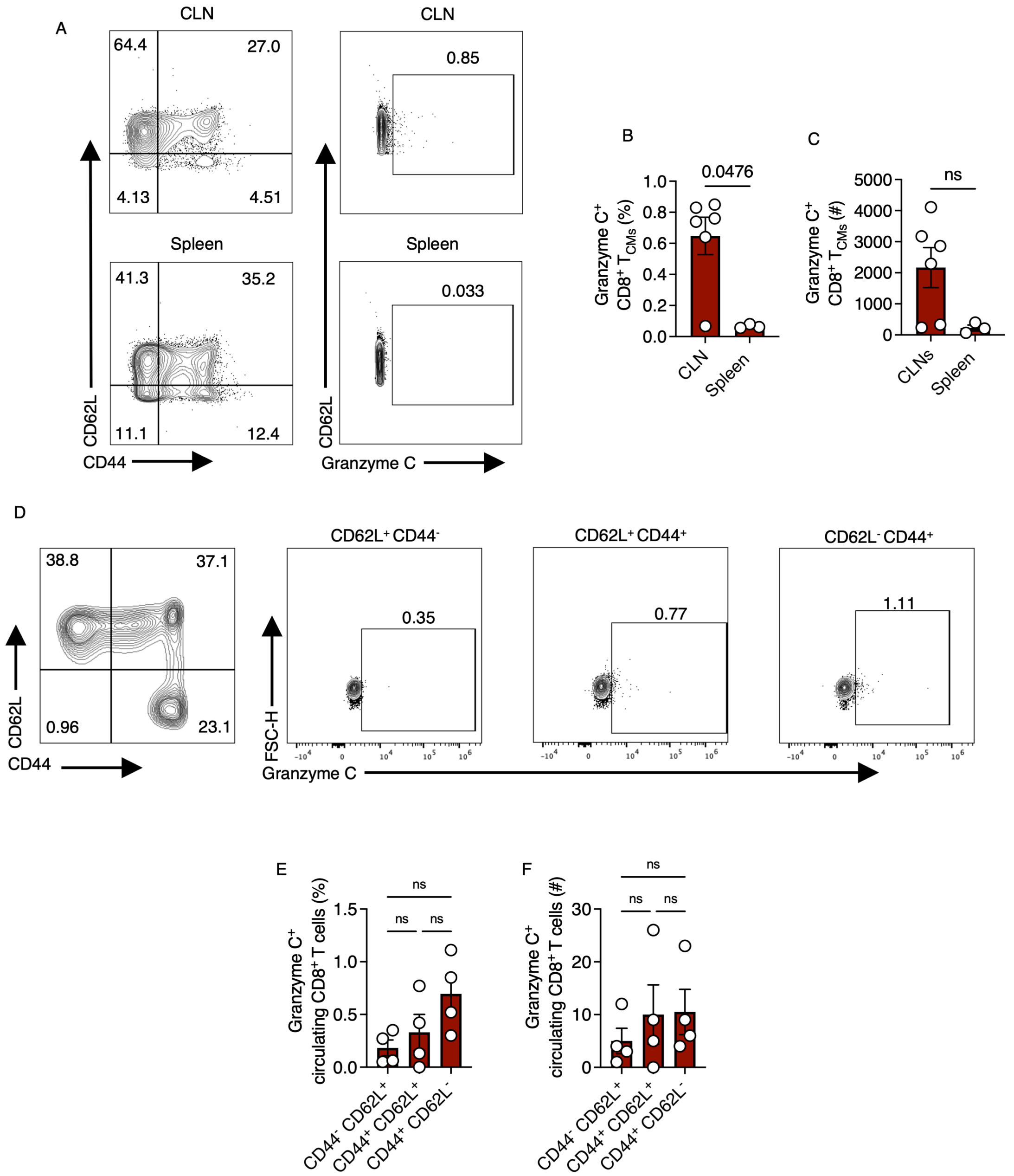

Supplementary Figure 3. Circulatory and lymph node-resident memory CD8+ T cells express little granzyme C.

(**A**) Flow cytometry plots of granzyme C expression by CD8+ T_{CMs} isolated from the cervical lymph nodes (CLNs) and spleens of sex- and age-matched C57BL/6 mice at least 28 dpi with VACV-SIINFEKL. CD8+ T_{CMs} were gated as CD45+ CD8α+ CD62L+ CD44+. (**B-C**) Percentages (B) and number (C) of granzyme C+ CD8+ $T_{CMs.}$ This experiment was performed 3 times with 3 mice/experiment. One representative experiment is shown. Dots represent individual CLNs/spleens. Error bars show the SEM. Statistics = Man-Whitney tests. (**D**) Flow cytometry plots of the gating strategy used to identify circulating granzyme C+ CD8+ T cell subsets based on CD44 and CD62L

expression from lymphocytes isolated from whole blood of C57BL/6 mice at least 28 dpi with VACV-SIINFEKL. Circulatory $CD8^+$ T cells were initially defined as $CD45^+$ $CD3^+$ $CD8\beta^+$. (**E-F**) Percentages (E) and numbers (F) of circulatory granzyme C^+ $CD8^+$ T cell subsets based on CD44 and CD62L expression . Data representative of 2 independent experiments with 4 mice/group . Dots represent individual ears. Error bars show the SEM. Statistics = Kruskal-Wallis tests.

Figure S4.

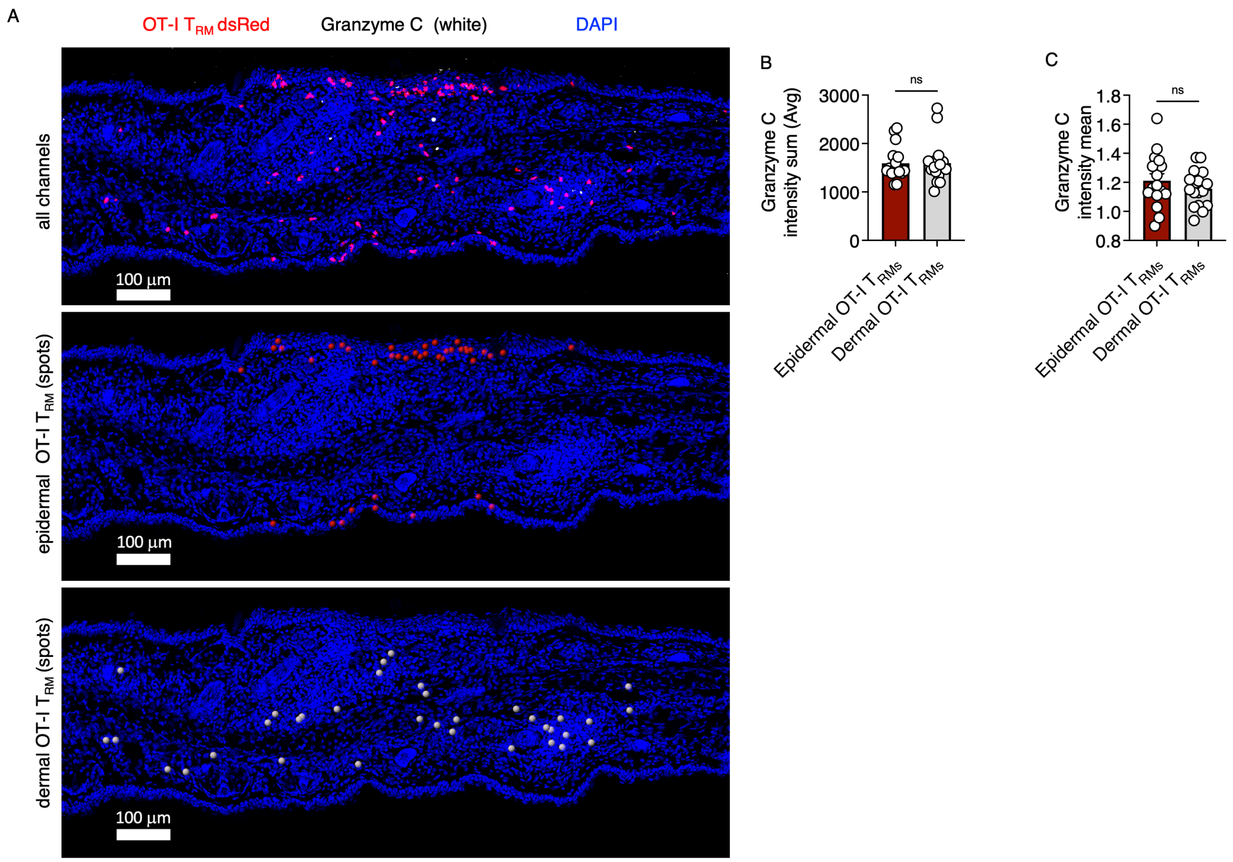

Supplementary Figure 4. Similar granzyme C expression in epidermal and dermal OT-I $CD8^+$ T_{RM} cells.

(**A**) Confocal images of frozen cross-sections of ear skin of *Cd8a*$^{-/-}$ mice that received 1 x 10^4 dsRed OT-I $CD8^+$ T cells prior to epicutaneous infection with VACV-NP-S-eGFP (containing SIINFEKL). Images were acquired at 28 dpi. Top panel displays all fluorescent channels (colors). Middle panel shows algorithm-classified spots for epidermal OT-I $CD8^+$ T_{RM} cells (red spots). Bottom panel shows algorithm-classified spots for dermal OT-I $CD8^+$ T_{RM} cells (gray spots). Scale bars represent 100 µm. Images taken from 16 sections from 2 mice. (**B-C**) Average intensity sums (B) and intensity mean

(C) of granzyme C between epidermal and dermal OT-I CD8$^+$ T cells identified using spots algorithm. Dots represent individual images. Error bars = SEM. Statistics = Mann-Whitney U tests.

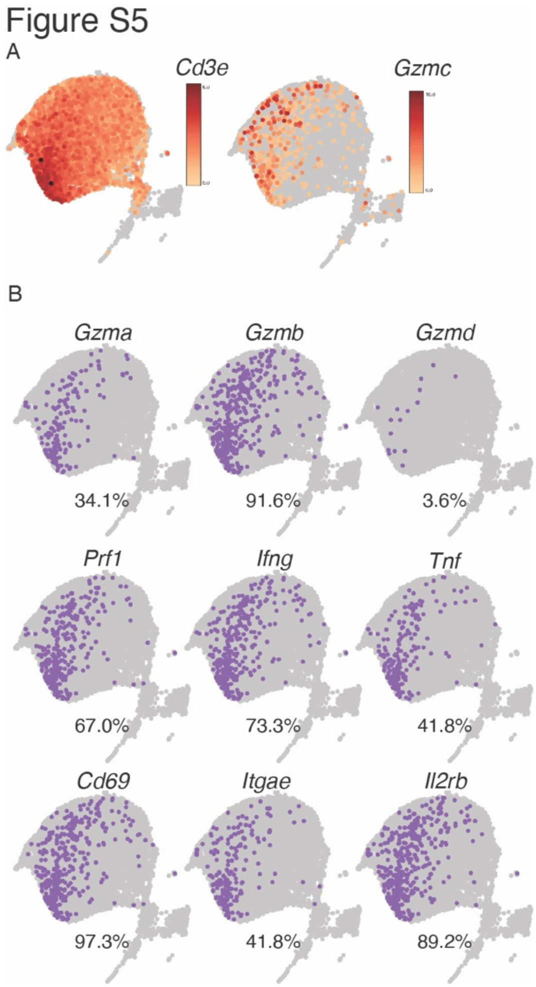

Supplemental Figure 5. OT-I CD8$^+$ T$_{RM}$ express *Gzmc* mRNA in the skin.

(**A**) UMAP of scRNA-seq data from OT-I CD8$^+$ T$_{RM}$ cells and T-bet$^+$ ILC1s sorted from the skin of Rag1-/- Tbet-Zgreen mice that had received 1 x 10^4 OT-I CD8$^+$ T cells prior to infection with VACV-SIINFEKL. Cells were sorted >28 days post infection. Left panel shows expression of Cd3e mRNA and right shows Gzmc (granzyme C) mRNA. (**B**) Overlay of cells expressing the indicated genes in *Cd3e*$^+$ *Gzmc*$^+$ cells. Purple dots indicate positive cells. Percentages indicate the percentage of cells positive for each gene.

Figure S6.

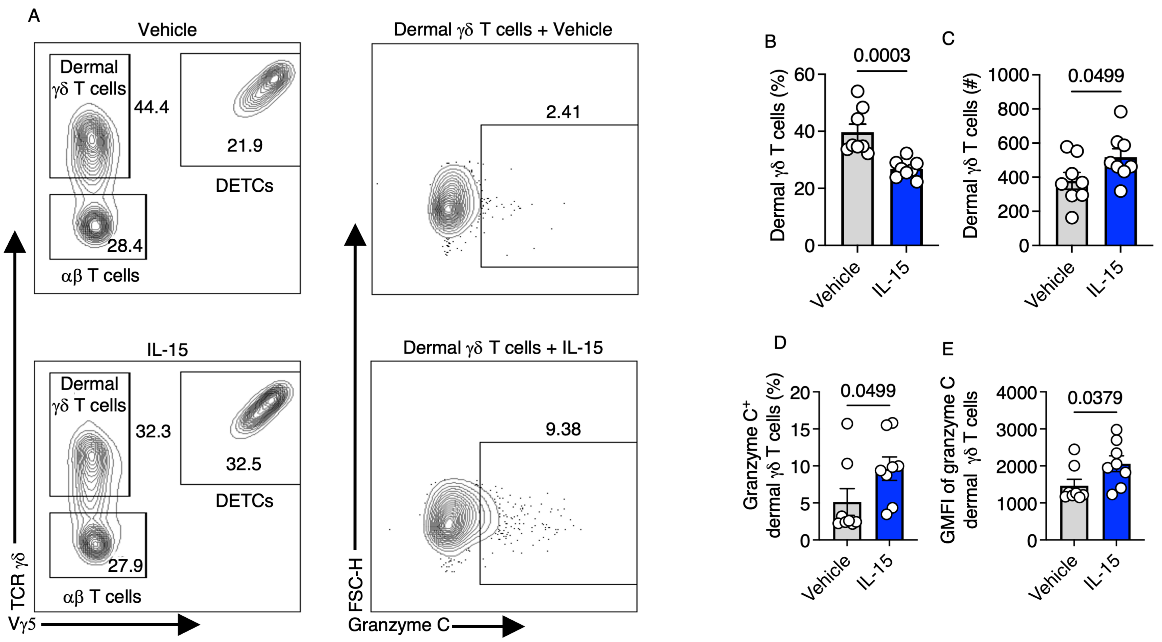

Supplementary Figure 6. Dermal γδ T cells upregulate granzyme C after IL-15 treatment.

(**A**) Flow cytometry plots of cutaneous T cells isolated from naïve C57BL/6 mice either treated with IL-15 or vehicle control. Cells were gated on $CD45^{+}$ CD45.2 IV^{-} $CD3^{+}$. Dermal γδ T cells were gated on $CD45^{+}$ CD45.2 IV^{-} $CD3^{+}$ $TCR\gamma\delta^{+}$ $V\gamma5^{-}$. (**B-C**) Frequencies (B) and numbers (C) of dermal γδ T cells isolated from ear pinna of C57BL/6 mice either treated with IL-15 or vehicle control. (**D-E**) Frequencies and MFIs of granzyme C in dermal γδ T cells between treatment groups. Data in B-E are representative of 2 independent experiments with 4 mice/group. Dots represent individual ears. Error bars show the SEM. Statistics = Mann-Whitney tests.
